# Failure rates in surgical treatment in adults with bacterial arthritis of a native joint: a systematic review of 8,586 native joints

**DOI:** 10.1007/s00402-023-04958-z

**Published:** 2023-07-03

**Authors:** Alex B. Walinga, Tobias Stornebrink, Kaj S. Emanuel, Arthur J. Kievit, Stein J. Janssen, Gino M. M. J. Kerkhoffs

**Affiliations:** 1 Department of Orthopedic Surgery and Sports Medicine, Amsterdam UMC Location University of Amsterdam, Amsterdam, The Netherlands; 2 Amsterdam Movement Sciences, Sport, Musculoskeletal Health, Amsterdam, The Netherlands; 3 Academic Center for Evidence Based Sports Medicine (ACES), Amsterdam, The Netherlands; 4 Amsterdam Collaboration for Health and Safety in Sports (ACHSS), International Olympic Committee (IOC) Research Center Amsterdam UMC), Amsterdam, The Netherlands; 5 Department of Orthopedic Surgery, CAPHRI Care and Public Health Research Institute, Maastricht University Medical Center+, Maastricht, The Netherlands

**Keywords:** Bacterial arthritis, Septic arthritis, Failure rate, Risk factors, Prognostic, Debridement, Reintervention, Reoperation, Reinfection

## Abstract

**Introduction:**

Most adult cases of bacterial–septic–arthritis of a native joint are effectively managed with a single surgical debridement, but some cases may require more than one debridement to control the infection. Consequently, this study assessed the failure rate of a single surgical debridement in adults with bacterial arthritis of a native joint. Additionally, risk factors for failure were assessed.

**Materials and Methods:**

The review protocol was registered on PROSPERO (CRD42021243460) before data collection and conducted in line with the ‘Preferred Reporting Items for Systematic Reviews and Meta-Analyses’ (PRISMA) guidelines. Multiple libraries were systematically searched to identify articles including patients reporting on the incidence of failure (i.e. persistence of infection requiring reoperation) of the treatment of bacterial arthritis. The quality of individual evidence were assessed using the Quality in Prognosis Studies (QUIPS) tool. Failure rates were extracted from included studies and pooled. Risk factors for failure were extracted and grouped. Moreover, we evaluated which risk factors were significantly associated with failure.

**Results:**

Thirty studies (8,586 native joints) were included in the final analysis. The overall pooled failure rate was 26% (95% CI 20 to 32%). The failure rate of arthroscopy and arthrotomy was 26% (95% CI 19 to 34%) and 24% (95% CI 17 to 33%), respectively. Seventy-nine potential risk factors were extracted and grouped. Moderate evidence was found for one risk factor (synovial white blood cell count), and limited evidence was found for five risk factors (i.e. sepsis, large joint infection, the volume of irrigation, blood urea nitrogen-test, and blood urea nitrogen/creatinine ratio).

**Conclusion:**

A single surgical debridement fails to control bacterial arthritis of a native joint in approximately a quarter of all adult cases. Limited to moderate evidence exists that risk factors associated with failure are: synovial white blood cell count, sepsis, large joint infection, and the volume of irrigation. These factors should urge physicians to be especially receptive to signs of an adverse clinical course.

**Supplementary Information:**

The online version contains supplementary material available at 10.1007/s00402-023-04958-z.

## Introduction

Bacterial arthritis, also known as septic arthritis, of a native joint is a clinical emergency that requires prompt treatment [1–4]. Most patients are effectively managed with a single surgical debridement of the joint in combination with systemic antibiotics, but some cases may require more than one debridement to control the infection.

Reported failure rates of a single surgical debridement vary widely [5, 6], and a structured assessment of risk factors for failure is lacking. Identification of risk factors may help to create a more uniform and evidence-based treatment approach to bacterial arthritis in clinical practice, which now relies predominantly on convention and local preferences [7, 8].

Consequently, the purpose of this systematic review is to assess the failure rate of a single –arthroscopic or open– surgical debridement in adults with bacterial arthritis of a native joint and to identify risk factors (e.g. demographics, medical history, lab markers, immunosuppression, and initial treatment approach) for failure of a single surgical debridement.

## Materials and methods

### Study design

The review protocol was registered on PROSPERO (CRD42021243460) before data collection. This study was conducted in line with the ‘Preferred Reporting Items for Systematic Reviews and Meta-Analyses’ (PRISMA) guidelines [9].

In consult with a medical librarian, we searched eligible studies using the PubMed, Embase, and Cochrane libraries between January 1980 and January 2021 using the following keywords: “bacterial arthritis”, “treatment failure”, “native joint”, and “risk factors”, including synonyms, related words, and MeSH Terms (appendix A).

Studies were included if they included 10 or more patients (16 years or older) with bacterial arthritis of a native appendicular joint, who underwent either open or arthroscopic surgical debridement. Furthermore, the studies had to report the incidence of failure (i.e. persistence of infection requiring reoperation or mortality) of the treatment. We excluded studies that did not present original data, meeting abstracts, case reports, animal or cadaveric studies, studies reporting on arthroplasty or with a foreign body in the affected joint (e.g. anchor), and studies published in a language other than English.

Two reviewers (AW, TS) independently screened titles and abstracts for eligibility using predefined criteria. Subsequently, full texts of selected papers were obtained and screened. Discordant judgment in study selection was resolved by consensus discussion together with a third reviewer (SJ). Bibliographies of included studies were screened to assess whether eligible studies were missed by our search.

Data were extracted using Microsoft Excel v. 16.52 (Microsoft Inc., Redmond, WA, USA).

Two authors (AW, TS) assessed the risk of bias independently and the Quality in Prognostic Studies (QUIPS) tool was used [10]. The QUIPS tool guides quality assessment in 6 domains: study participation, study attrition, prognostic factor measurement, outcome measurement, study confounding, and statistical analysis and reporting. The risk of bias is reported as low, moderate, or high for each domain and then an overall risk of bias is assigned based on the ratings in each domain (appendix B1-B2).

### Variables and outcome measures

The primary outcome variable was the rate of failure. Additionally, the following variables were extracted per study: year of publication, country, study type, years of inclusion, number of patients and joints with bacterial arthritis, sex distribution, age, microorganism profile, surgical technique, number of total operations, and potential risk factors associated with failure of a single surgical debridement.

We also extracted the criteria used to define failure of a single surgical debridement.

### Statistical analysis

We performed a meta-analysis by pooling the failure rate from individual studies using a random-effects model with an inverse variance method and logit transformation. 95%-confidence intervals were assessed using the Clopper-Pearson interval. We separately analysed the failure rate of arthroscopy and arthrotomy, and the failure rate of shoulder and knee bacterial arthritis to increase homogeneity. Heterogeneity among studies was evaluated by calculating I^2^ and a chi-square Q test [11]. A p-value of < 0.05 was considered to indicate significant heterogeneity. Analyses were conducted using the meta package in R version 3.04 (R studio, Northern Ave, Boston, USA) [12].

### Best-evidence-synthesis

The strength of the evidence for risk factors was evaluated using a best-evidence synthesis in absence of reported effect sizes, which did not allow pooling of data. Only the risk factors with significant association in at least one included study were categorized. The strength of evidence for each identified risk factor was based on the guidelines by Furlan et al. [13], additional details on the guidelines are provided in Appendix B.

The overall risk of bias was reported as low, moderate, or high. Studies reported as low risk of bias were categorized as high-quality studies, and studies reported as moderate and high risk of bias were reported as low-quality studies).

## Results

### Study characteristics and quality appraisal

**Fig. 1:**
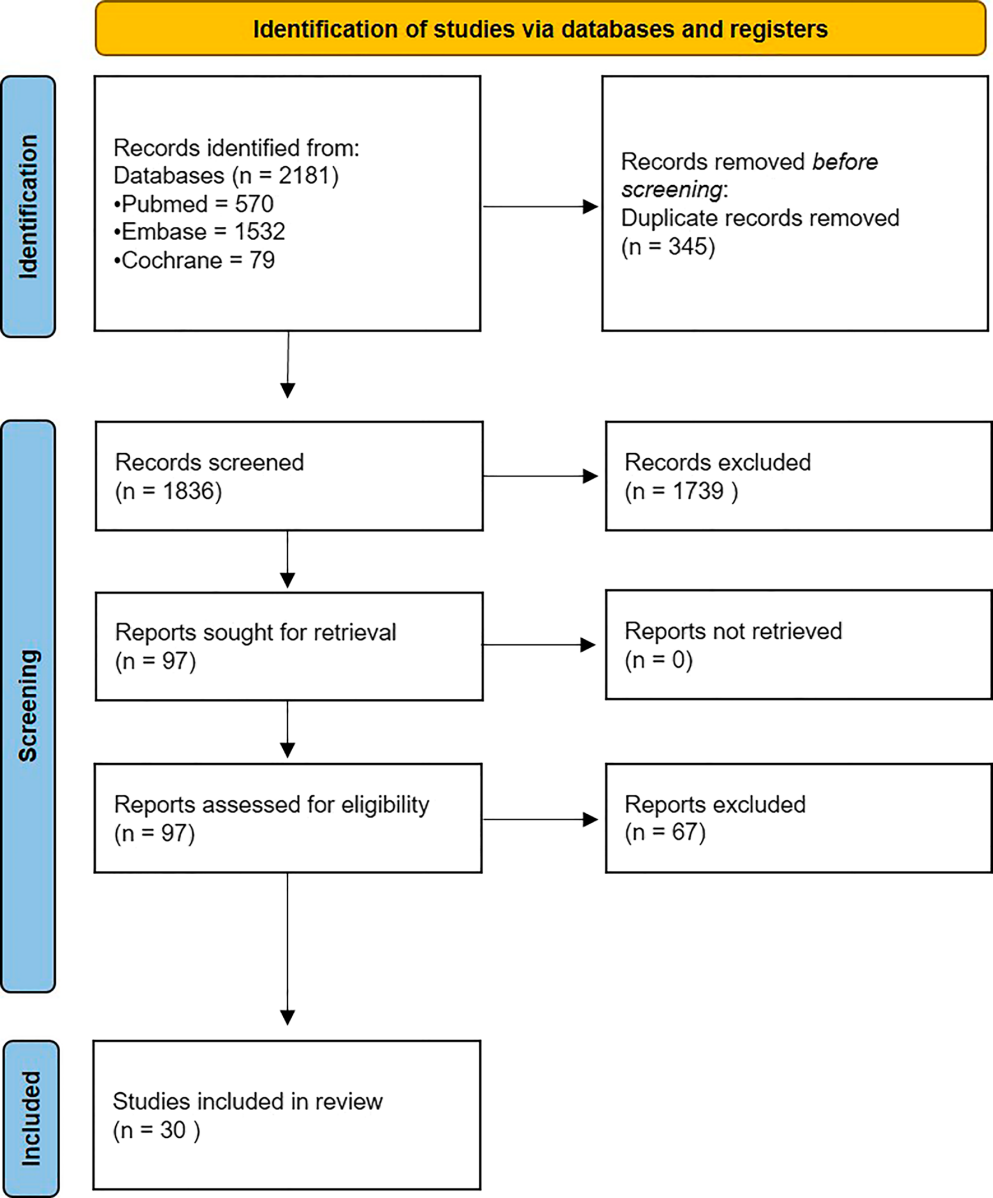
This PRISMA flowchart demonstrates the number of papers identified and the article selection using predefined eligibility criteria. *N*  number of papers

The search yielded 1,836 potentially eligible studies (Fig. 1). After screening titles and abstracts, 1,739 studies were excluded. Subsequent full-text screening resulted in 30 studies that were included in this review. Twenty-nine studies (97%) had a retrospective design. The majority of the included studies (n = 26, 87%) were published after 2010.

**Fig. 2:**
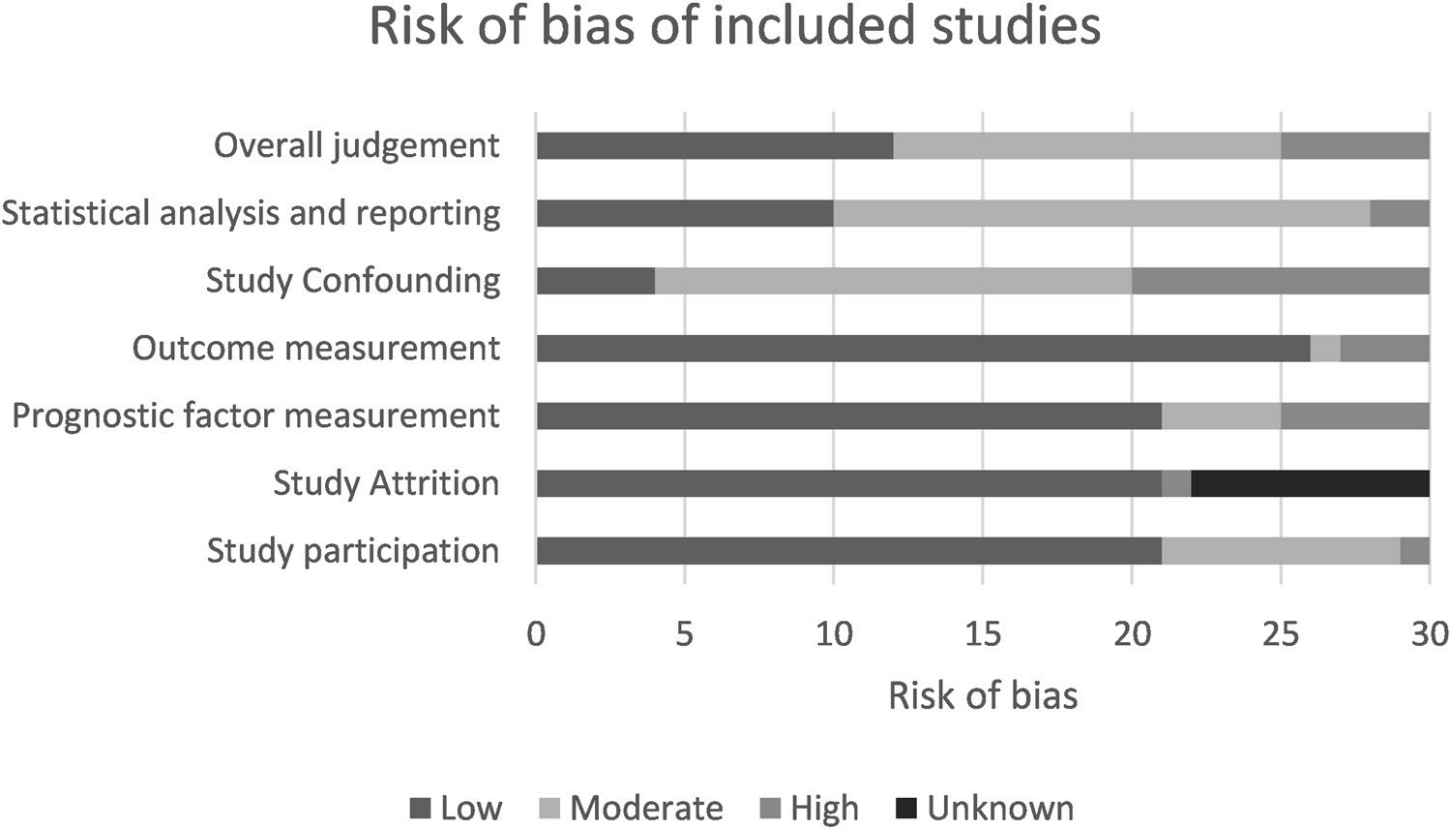
Risk of bias of included studies using the QUIPS-tool

Twelve of the studies [5, 14–24] were at low risk of bias, five [6, 25–28] were at high risk of bias and 13 [29–41] were at moderate risk of bias (Fig. 2 and Appendix B1-B2).

### Patient characteristics

The 30 studies reported on a total of 8,569 patients with bacterial arthritis of 8,586 native joints. The average age of patients was 59 years (range: 42 to 72 years) and 61% were men (range: 31 to 89%; Table 1).

**Table 1:** Study characteristics per included paper

Author	Year	Country	Study type	Year included	N. of patients	N. of joints	N. of males (%)	Mean age (range)	N. of operations	N. of arthroscopy	N. of arthrotomy	N. of failure rate (%)
Abdou M.A et al. [24]	2019	Korea	Retrospective	2004–2015	36	36	18 (50)	63 (38–82)	36	36	0	7 (19)
Aim F et al. [25]	2014	France	Retrospective	NR	46	46	29 (63)	46 (18–71)	46	46	0	13 (28)
Al-Nammari S et al. [5]	2007	United Kingdom	Retrospective	2000–2005	48	48	43 (74)	60 (NR)	48	45	3	4 (8)
Assuncão J.H. et al. [14]	2018	Brazil	Retrospective	2004–2014	27	27	12 (44)	46 (NR)	27	0	27	8 (30)
Besnard M et al. [15]	2018	France	Retrospective	2000–2013	52	52	33 (63)	62 (51–78)	52	52	0	17 (33)
Bohler C et al. [29]	2016	Austria	Retrospective	2002–2010	70	70	46 (66)	59 (36–72)	70	41	29	8 (11)
Bohler C et al. [30]	2017	Austria	Retrospective	2001–2015	59	59	25 (43)	72 (57–82)	59	21	38	18 (31)
Bovonratwet P et al. [16]	2019	USA	Retrospective	2005–2016	255	255	156 (61)	64 (NR)	255	155	100	30 (12)
Bovonratwet P et al. [17]	2017	USA	Retrospective	2005–2014	384	384	258 (67)	59 (NR)	384	216	168	62 (16)
Cho C.H et al. [31]	2015	Korea	Retrospective	2006–2021	32	34	15 (47)	62 (32–79)	34	22	12	5 (15)
Faour M et al. [26]	2018	USA	NR	2011–2015	695	695	452 (65)	59 (NR)	695	464	231	78 (11)
Hunter J.G et al. [18]	2015	USA	Retrospective	2000–2011	128	132	82 (64)	55 (NR)	132	42	90	50 (38)
Jaffe D et al. [32]	2016	USA	Retrospective	2000-NR	80	80	48 (60)	54 (43–63)	80	33	47	17 (21)
Jeon I et al. [27]	2006	South Korea	Retrospective	2001–2004	19	19	17 (89)	59 (23–85)	19	19	0	5 (26)
Jiang J.J et al. [33]	2017	USA	Retrospective	2002–2011	5154	5154	3151 (61)	61 (NR)	5154	799	4355	637 (12)
Johns, B.P et al. [19]	2017	Australia	Retrospective	2000–2015	161	166	108 (67)	62 (NR)	166	123	43	90 (54)
Joo, Y.B et al. [20]	2019	Korea	Retrospective	2007–2019	97	97	53 (55)	61 (21–97)	97	97	0	12 (12)
Jung, S.W et al. [21]	2017	Korea	Retrospective	2005–2014	137	137	60 (44)	66 (23–90)	137	126	11	44 (32)
Kang T et al. [34]	2018	South Korea	Retrospective	2013–2016	55	55	17 (31)	66 (26–86)	55	55	0	16 (29)
Kao F.C et al. [35]	2019	Taiwan	Retrospective	2005–2016	51	51	32 (63)	59 (28–94)	51	NR	NR	5 (10)
Khazi Z.M et al. [36]	2019	USA	Retrospective	2007–2017	421	421	222 (53)	NR	421	34	387	191 (45)
Khazi Z.M. et al. [37]	2020	USA	Retrospective	2006–2016	204	204	133 (65)	NR	204	147	57	24 (12)
Kuo C.L et al. [6]	2011	Taiwan	Retrospective	1996–2008	39	39	33 (85)	42 (18–73)	39	39	0	28 (72)
Lee D.K et al. [38]	2019	Korea	Retrospective	2001–2015	57	57	31 (54)	56 (20–87)	57	27	30	20 (35)
Mabille C et al. [39]	2020	France	Retrospective	2010–2017	46	46	32 (70)	60 (46–68)	46	23	23	14 (30)
Matsuhashi T et al. [28]	2011	Japan	Retrospective	1992–2005	10	10	4 (40)	62 (25–86)	10	10	0	1 (10)
Rhee S.M et al. [40]	2020	Korea	Retrospective	2001–2015	31	31	12 (39)	55 (22–76)	31	31	0	17 (55)
Sammer D.M et al. [22]	2009	USA	Retrospective	1997–2007	36	40	13 (36)	63 (25–89)	40	21	19	19 (48)
Stake S et al. [23]	2020	USA	Retrospective	2005–2015	63	63	45 (71)	53 (NR)	63	53	10	18 (34)
Stutz G et al. [41]	2000	Switzerland	Retrospective	1988–1998	76	78	44 (56)	53 (17–94)	78	78	0	33 (42)

*NR*  not reported, *N* number

Bacterial arthritis was most commonly reported in the shoulder (70%), followed by the knee (21%) (Appendix C). The most commonly identified microorganism was Staphylococcus Aureus (44%) (Table 2). Of the 8,586 surgical debridements, 5,680 (66%) were performed via an arthrotomy and 2,855 (33%) arthroscopically. One study (n = 51, 1%) did not report the surgical approach [35].

**Table 2:** Number per identified microorganism

Causative organisms	Total number of cases
**Staphylococcus ssp.**	368
Staphylococcus Aureus	281
- Methicillin-Resistant Staphylococcus Aureus	72
- Methicillin-Susceptible Staphylococcus Aureus	138
- Staphylococcus Aureus not specified	71
Coagulase-negative Staphylococcus	18
Other subtypes of Staphylococcus	55
Uknown Staphylococcus ssp.	14
**Streptococcus ssp**.	54
Group-B-Streptococcus	8
Streptococcus Pneumoniae	5
Other subtypes of Streptococcus	13
Unknown Streptococcus ssp.	28
**Escherichia Coli**	14
**Pseudomonas Aeruginosa**	21
**Other**	47
**Not reported or specified (n = 12 studies)**	8,082
**Culture positive**	721*
**Culture negative**	311

*n* number

*This amount is greater than the sum of all reported organisms since some studies did not specify the positive cultures

### The failure rate

**Fig. 3:**
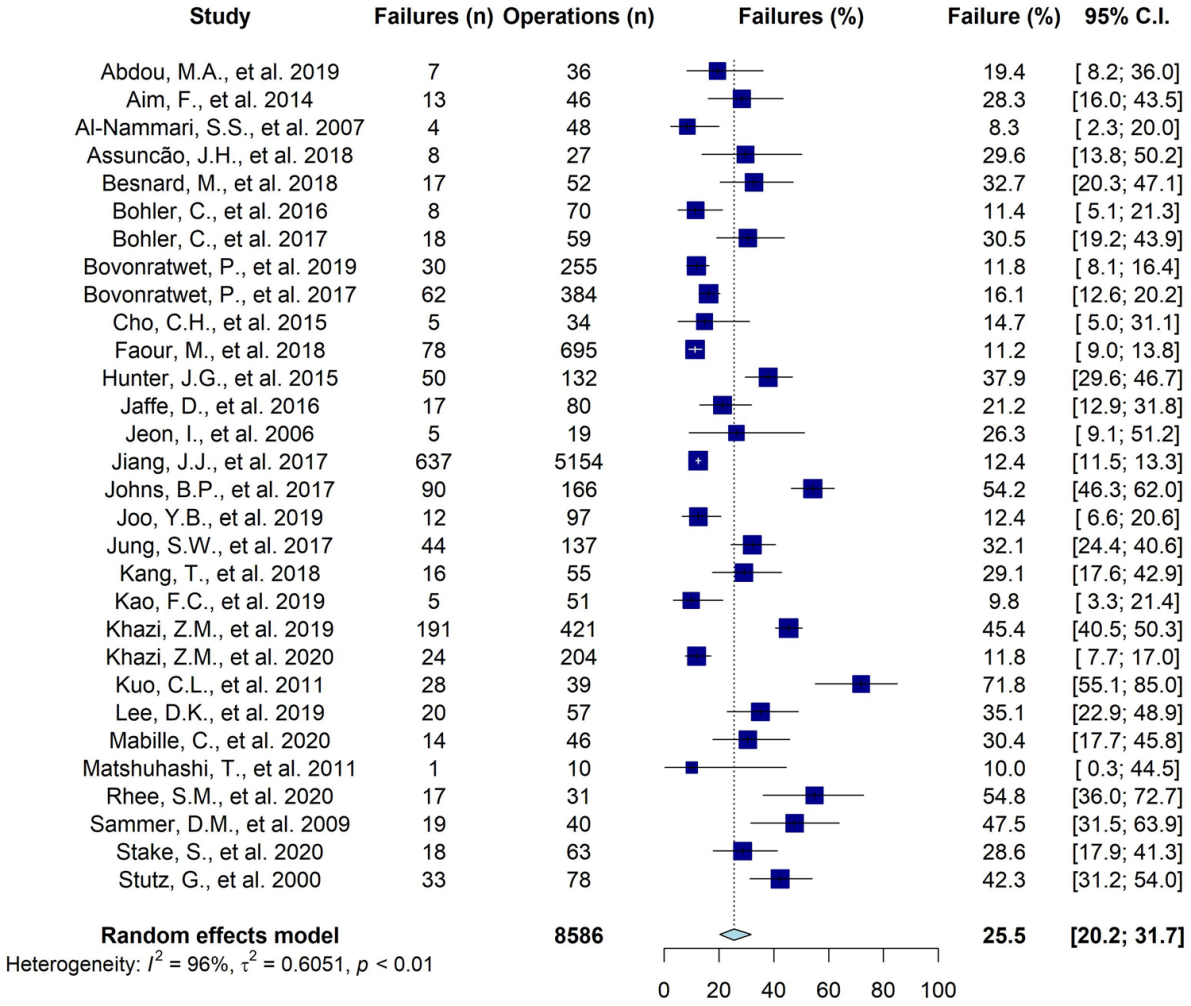
Forest plot of the overall failure rate**.** N number, C.I. Confidence interval

The overall pooled failure rate of a single surgical debridement was 25.5% (range among studies: 8.3% to 72% (95% CI 20 to 32%, Fig. 3). The heterogeneity was high with I^2^ = 96% (p < 0.01). Therefore, additional analyses were conducted in an attempt to parse out this heterogeneity. The study of Jiang et al. [33] (5154 shoulders, with a failure rate of 12.4%) had a relatively high weight of 60% in the model. However, the removal of the study from the model did not affect the pooled failure rate (26.2%; 95% CI 21 to 33%, I^2^ = 93%).

**Fig. 4:**
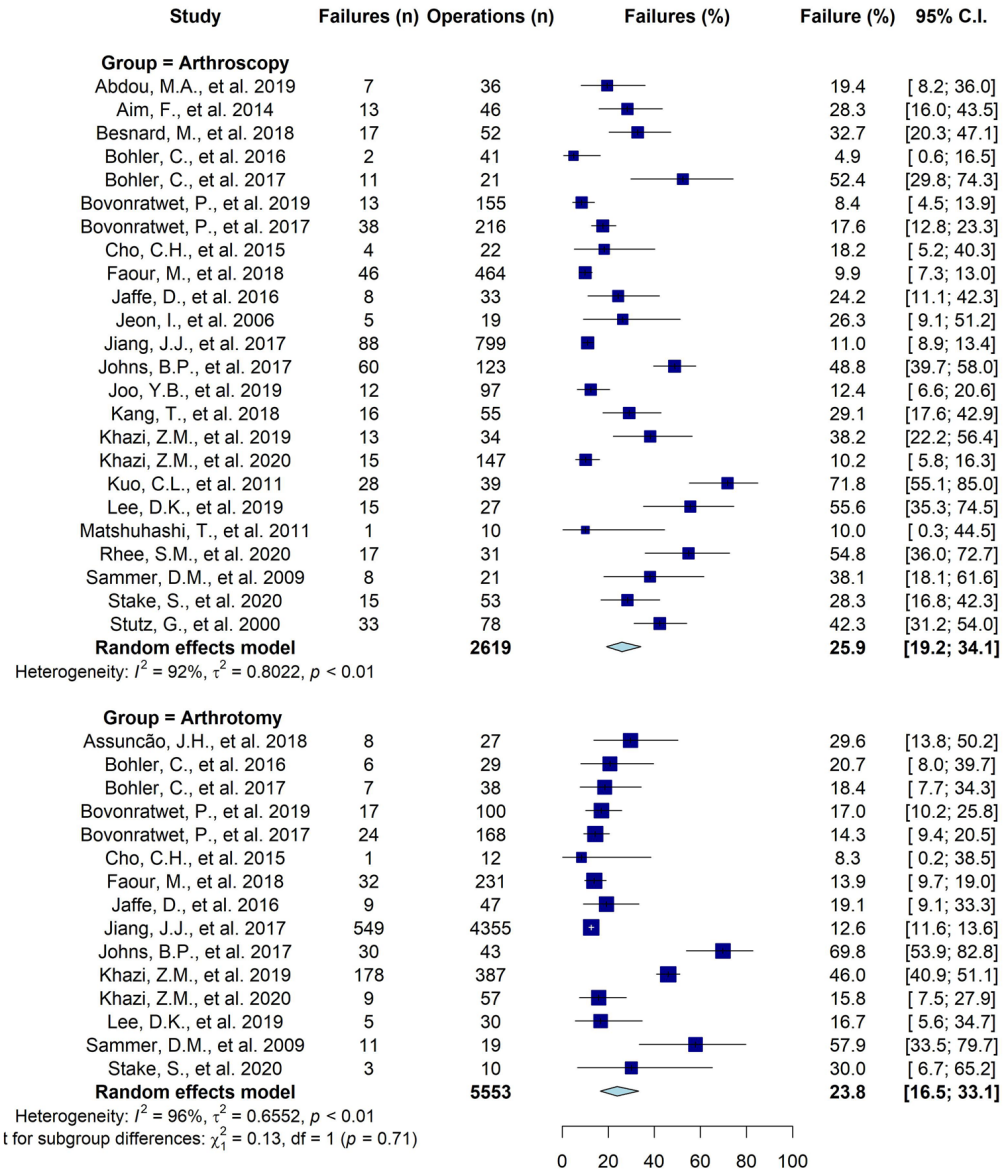
Forest plot of the failure rate for arthroscopy and arthrotomy**.** N  number, C.I.  Confidence interval

A sub-group analysis of the failure rate of the separate surgical approaches, which was reported in 25 studies, showed a failure rate of arthroscopy of 26% (95% CI 19 to 34%, I^2^ = 92%, ranging from 4.9 to 72%, Fig. 4), and a failure rate of arthrotomy of 24% (95% CI 17 to 33%, I^2^ = 96%, ranging from 8.3% to 70%). Thirteen studies specifically compared arthroscopy and arthrotomy as potential risk factors. Four out of thirteen (31%) studies identified arthrotomy as a statistically significant risk factor for failure of a single debridement. In contrast, two out of these thirteen (15%) studies identified arthroscopy as a significant risk factor (Table 3).

**Table 3:** Potential risk factors for failure of single surgical debridement

Prognostic factor	Number of patients	Yes	No	High quality +	High quality −	Low quality +	low quality −	Level of evidence
Sepsis	558	2	0	1 [21]		1 [36]		Limited +
Synovial white blood cell count	368	3	1	2 [18, 23]	1 [20]	1 [32]		Moderate +
Staphylococcus aureus infection	284	2	1	1 [18]	1 [20]	1 [30]		Conflicting
Concurrent infection	242	2	1	1 [23]	1 [18]	1 [35]		Conflicting
Large joint infection	128	1	0	1 [18]				Limited +
Volume of irrigation	97	1	0	1 [20]				Limited +
ASA-classification	80	1	0			1 [32]		Inconclusive
Blood urea nitrogen (BUN) test	63	1	0	1 [23]				Limited +
BUN/creatinine ratio	63	1	0	1 [23]				Limited +
Erosions seen in MRI but not in X-ray	57	1	0			1 [38]		Inconclusive
Erosions seen in both x-ray and MRI	57	1	0			1 [38]		Inconclusive
History of liver cirrhosis	51	1	0			1 [35]		Inconclusive
Presence of comorbidities	442	3	3	1 [19]		2 [30, 34]	3 [25, 29, 35]	Conflicting
MRSA-infection	353	2	2	1 [18]	2 [5, 20]	1 [32]		Conflicting
Race	191	1	1	1 [23]	1 [18]			Conflicting
Hospitalization	129	1	1			1 [29]	1 [30]	Conflicting
Gächter stage III—IV	105	1	1			1 [25]	1 [30]	Conflicting
Arthrotomy	7639	4	9	3 [16, 19, 22]	2 [17, 23]	1 [29]	7 [26, 30, 32, 33, 36–38]	Conflicting
Arthroscopy	7639	2	11		5 [16, 17, 19, 21, 23]	2 [30, 38]	6 [26, 29, 32, 33, 36, 37]	Strong -
Positive synovial fluid culture	509	3	4	2 [19, 24]	2 [20, 23]	1 [25]	2 [34, 35]	Conflicting
Positive synovial fluid gram stain	246	1	2		2 [18, 23]	1 [34]		Conflicting
Gender	1151	1	9		4 [18–20, 23]	1 [29]	5 [25, 30, 34–36]	Strong -
Serum white blood cell count	417	2	3	2 [18, 23]	1 [20]		2 [29, 30]	Conflicting
History of immunosuppression	243	1	2	1 [15]	2 [18, 23]			Conflicting
Inflammatory arthritis	243	1	2	1 [18]	2 [15, 23]			Conflicting
Diabetes Mellitus	468	1	5	1 [18]	2 [20, 23]		3 [29, 30, 35]	Moderate -

‘ + ’ = positive as risk factor, ‘− ‘ = negative for risk factor, [**] = reference, *ASA* American society of anaesthesiologists classification, *MRSA* methicillin-resistant staphylococcus aureus

A sub-group analysis of the failure rate of the shoulder, which was reported in 11 studies, showed an overall failure rate of 19% (95% 13 to 26%, I^2^ = 84%, ranging from 8.3 to 56%, appendix D). The failure rate of shoulder arthroscopy was 22% (95% CI 13 to 34%, I^2^ = 89%, ranging from 8.4 to 56%), and for arthrotomy it was 14% (95% CI 12 to 17%, I^2^ = 0%, ranging from 8.3 to 18%). A sub-group analysis of the failure rate of the knee, which was reported in eight studies, showed an overall failure rate of 26% (95% CI 17 to 44%, I^2^ = 93%, ranging from 4.9 to 72%, appendix D). The failure rate of knee arthroscopy was 26% (95% CI 14 to 44%, ranging from 4.9 to 72%), and for arthrotomy it was 25% (95% CI 13 to 44%, ranging from 14 to 70%). The failure rate of the hip was reported in three studies and showed a failure rate of 10% (n = 51), 45% (n = 421), and 53% (n = 17). Only one of these studies reported the failure rate of arthroscopy (38%) and arthrotomy (46%), separately.

### Risk factors for failure of single surgical debridement

The included studies investigated 79 different factors for association with failure. Twenty-six of these factors were found to be a statistically significant predictor of failure of a single debridement in one or more studies (Table 3). Based on the best-evidence-syntheses of risk factors for failure, there were no positive risk factors classified as strong evidence, one risk factor (i.e. synovial white blood cell count) classified as moderate evidence, and five risk factors (i.e. systemic sepsis, large joint infection, the volume of irrigation, blood urea nitrogen-test (BUN), and BUN/creatinine ratio) were classified as limited evidence. Twelve risk factors were classified as conflicting evidence and four as inconclusive evidence (Table 3 and Appendix C).

### Definitions of failure of single surgical debridement (Table 4)

**Table 4:** Definitions of failure of single debridement of bacterial arthritis requiring reoperation

Definition	N. of studies	References
Clinical findings, laboratory signs of systemic inflammation, and positive/purulent fluid analysis	7	[18, 20, 21, 23–25, 30]
Clinical findings and laboratory signs of systemic inflammation	7	[5, 6, 19, 27, 31, 38, 40]
Clinical findings and positive/purulent fluid analysis	1	[22]
Recurrence of infection (not further specified)	5	[15, 29, 32, 35, 41]
Reported failure with unclear definition	6	[16, 17, 28, 33, 34, 39]
Missing	4	[14, 26, 36, 37]

*N* number

The most commonly used definition of failure was a combination of clinical findings, elevated laboratory signs of systemic inflammation, and/or positive/purulent fluids analysis requiring reoperation (n = 14, 47%).

## Discussion

The main finding of this study is that bacterial arthritis of a native joint can be treated successfully by a single surgical debridement in combination with systemic antibiotic therapy in the majority of patients. However, one should be vigilant, as in about a quarter, additional or repeated treatment is necessary to control the infection. Based on the best-evidence-synthesis; synovial white blood cell count, systemic sepsis, large joint infection, the volume of irrigation, blood urea nitrogen test, and blood urea nitrogen/creatinine ratio can be considered as risk factors for failure with limited to moderate evidence.

A wide range of failures from 8 to 72% was found in the included studies. The three studies with the lowest score in the risk of bias analyses and inclusion of several joints, Besnard et al., Hunter et al., and, Jung et al. [15, 18, 21] showed that after a single surgical debridement, 33% to 38% of patients require a reoperation. This is slightly higher than the 95% confidence interval of the pooled overall failure rates, which could indicate underreporting in higher risk-of-bias studies.

Over the years, various treatments have been advocated for bacterial arthritis, including repeated non-operative needle aspiration, open synovectomy, and arthroscopy with or without synovectomy. Early and aggressive intervention is essential in eradicating the infection and preventing joint damage [42]. In our study, we encompassed a wide timeframe for the inclusion of studies, ranging from 1980 to 2021. It is worth noting that four studies included in our analysis had an inclusion period predating 2000. This factor raises the possibility of potential influence on the results due to subsequent advancements in techniques. Furthermore, arthroscopic techniques have gained popularity as an alternative to arthrotomy in the treatment of bacterial arthritis. They allow for minimally invasive joint lavage and debridement, effectively reducing bacterial load and improving treatment outcomes [43]. Moreover, needle arthroscopy, an even less invasive approach, has emerged as a promising option in the management of bacterial arthritis, providing visualization and irrigation of the infected area, under local anaesthesia, through the insertion of a small arthroscope [44, 45].

In terms of failure rate, our systematic review does not support one surgical approach (i.e., arthrotomy versus arthroscopy) over another. Also, there was no clear difference found in the reported risk factor analyses that included the surgical approach. However, it should be noted that (1) these results are likely affected by selection bias; i.e. a longstanding or more severely ill patient is perhaps more likely to undergo arthrotomy versus arthroscopy, and (2) this systematic review was not designed to directly compare arthroscopy and arthrotomy in terms of other outcomes. A recent systematic review that did focus on these outcomes found a significantly lower complication rate and duration of hospital stay after arthroscopy [46].

In the shoulder, we found a pooled failure rate of 19%. Memon et al.[47] found –in a systematic review in 2018 including 121 shoulders– a failure rate of 30%. This difference might be explained by the fact that they included all ages (including children), did not focus on isolated bacterial arthritis, and included case reports which are often exceptional cases that may relate to higher reoperation and/or persistent infection rates. Furthermore, large studies, including one study with 5,154 cases [33], were published recently and were additionally included in our review. Therefore, the results of this review probably better reflect the failure rate in adult patients with bacterial arthritis of the native shoulder joint.

In the knee, we found an overall pooled failure rate of 26% (26% for arthroscopy, and 25% for arthrotomy). In a systematic review and meta-analysis –including seven studies with 1,089 knees– by Panjwani et al. [48], a failure rate of 17% in the arthroscopy group and 22% in the arthrotomy group was described. The authors concluded that arthroscopy results in a significantly lower re-operation rate than arthrotomy [48]. These findings should be interpreted with care as to the earlier mentioned potential selection bias inherent to non-experimental retrospective studies. Our results contradict their conclusion. Besides, in a randomized clinical trial by Peres et al. [49], no significant difference was shown between arthrotomy and arthroscopy; albeit both groups were small. Highly-quality experimental (randomized) controlled studies are necessary to confirm superiority or non-inferiority. Such study should not only focus on reoperation rate, but also on Patient-Reported Outcomes Measures, functional outcomes, hospital stay, and complications.

In the hip, we found a failure rate ranging from 10 to 53%. Because only three studies focused on the hip, we abstained from data pooling. According to a recent systematic review (n = 25 patients), arthroscopy was found to be both safe and effective in treating bacterial hip arthritis; however, there was no superiority of arthroscopy over arthrotomy (or vice-versa) [50]. In our review, we analysed a study involving 421 patients, out of which 387 received an arthrotomy and 34 underwent an arthroscopy. This study’s findings indicate that patients with bacterial arthritis of the hip demonstrated comparable rates of short-term complications and reoperations [36]. Based on this limited evidence there is no preference for hip arthroscopy over arthrotomy (or vice-versa). This is an important finding as hip arthroscopy is challenging and requires specific expertise that is not always available. 

There were 26 risk factors associated with failure. None of these risk factors were classified as strong evidence. Six risk factors were classified as moderate or limited evidence: synovial white blood cell count, sepsis, large joint infection, the volume of irrigation, blood urea nitrogen (BUN) test, and BUN/creatinine ratio. The BUN test and BUN/creatinine ratio are tests that are not routinely used [51]. Besides, these two parameters were only investigated by one small retrospective study (n = 63) [23], and hence should be interpreted with care. Synovial white blood cell count, sepsis, large joint infection, and the volume of irrigation are associated with the severity of the infection and virulent organisms. Another interesting finding of this review is that commonly assumed risk factors such as specific micro-organisms (e.g., Methicillin-resistant Staphylococcus aureus (MRSA), but also rheumatoid arthritis, diabetes, and other comorbidities were not found to be associated with failure.

We found five different criteria that helped define failure of a single surgical debridement among included studies. A combination of clinical findings, laboratory signs of systemic inflammation, and/or positive/purulent fluid analyses was the most commonly used criterion to prompt additional intervention. Nonetheless, none of the studies described clear cut-off points for failure of a single debridement and it was presumably ultimately at the discretion of the treating physician, which is challenging due to the lack of good quality diagnostic tests [52].

### Limitations

This study has several limitations. First, it is limited by the quality of included studies, which were all prone in varying degrees to bias. Study confounding and prognostic factor measurement were the most commonly identified biases (Fig. 2). Second, most of the meta-analyses presented significant heterogeneity among included studies. This can be explained by a wide range of populations, joints, and surgical techniques between studies. Therefore, random-effects models and sub-analyses by surgical technique and affected joint were conducted. No major differences were found between surgical techniques. However, in the studies on the shoulder joint, slightly lower failure rates were found than in the knee. The differences in joints between studies may therefore have been a source of heterogeneity. Third, a wide variety in number and definition of risk factors made it impossible to pool the risk factors for failure. Future well-designed high-quality studies are merited to confirm these results. Fourth, in contrast to what we expected based on our clinical practice, the shoulder was the most commonly affected joint in this systematic review–- mainly driven by the study of Jiang et al. [33] that added 5154 shoulder joints to our study. A sensitivity analysis that excluded these shoulders did not affect the overall pooled failure rate. Fifth, it should be noted that this review included all joints of the appendicular skeleton; nevertheless, a mere 0.9% (78 joints) of the arthritis pertained to small joints, including: the wrist, elbow, and ankle. It is important to note that inclusion of these cases does not significantly impact the overall pooled failure rate. To clarify, whether all these joints had either failed or succeeded, the overall failure rate would have only increased or decreased by no more than 1%. Sixth, although we had a wide timeframe for inclusion of studies (1980–2021), we only found 4 studies with an inclusion period predating 2000, which may influence the results due to the subsequent advancements in technique. However, it is important to note that the majority of the studies included in this review (n = 26) had an inclusion period after 2000, and thus generally reflect current practice.

## Conclusion

In conclusion, this systematic review found that a single surgical debridement fails to control the infection in native joint bacterial arthritis in 26% of cases. No difference in failure rates was found between arthroscopy and arthrotomy. Limited to moderate evidence exists that risk factors associated with failure are synovial white blood cell count, sepsis, large joint infection, and the volume of irrigation. These factors should urge physicians to be especially receptive to signs of an adverse clinical course.

### Supplementary Information

Below is the link to the electronic supplementary material.

Supplementary file1 (DOCX 314 KB)

## Data Availability

Not applicable.
