# Supplementary material for: Failure rates in surgical treatment in adults with bacterial arthritis of a native joint: a systematic review of 8,586 native joints
Source: Arch Orthop Trauma Surg. 2023 Jul 3;143(11):6547–59. doi: 10.1007/s00402-023-04958-z (PMC10541340; doi:10.1007/s00402-023-04958-z)
Supplement: Supplementary file 1 — Supplementary file1 (DOCX 314 KB) [file 402_2023_4958_MOESM1_ESM.docx]

**Supplementary: Failure Rates in Surgical Treatment in Adults with Bacterial Arthritis of a Native Joint: A Systematic Review of 8,586 Native Joints**

**Authors:**

Alex B. Walinga, MD^1,2,3,4^, a.b.walinga@amsterdamumc.nl

Tobias Stornebrink, MD^1,2,3,4^, t.stornebrink@amsterdamumc.nl

Kaj S. Emanuel, MD, PhD^1,2,3,4,5^, k.s.emanuel@amsterdamumc.nl

Arthur J. Kievit, MD^1,2,3,4^, a.j.kievit@amsterdamumc.nl

Stein J. Janssen, MD, PhD^1,2,3,4^, steinjanssen@gmail.com

Gino M.M.J. Kerkhoffs, MD, PhD^1,2,3,4^, g.m.kerkhoffs@amsterdamumc.nl

**Institutions and departments**

1. Amsterdam UMC location University of Amsterdam, Department of Orthopedic Surgery and Sports Medicine, Amsterdam, The Netherlands
2. Amsterdam Movement Sciences, Sport, Musculoskeletal Health, Amsterdam, The Netherlands
3. Academic Center for Evidence based Sports medicine (ACES), Amsterdam, The Netherlands
4. Amsterdam Collaboration for Health and Safety in Sports (ACHSS), International Olympic Committee (IOC) Research Center Amsterdam UMC
5. Department of Orthopedic Surgery, CAPHRI Care and Public Health Research Institute, Maastricht University Medical Center+, Maastricht, the Netherlands

**Corresponding Author**

Alex B. Walinga, Department of Orthopedic Surgery and Sports Medicine, Amsterdam University Medical Center, University of Amsterdam, Meibergdreef 9, 1105AZ, Amsterdam, the Netherlands, T: +31205666540, Email: [a.b.walinga@amsterdamumc.nl](mailto:a.b.walinga@amsterdamumc.nl)

**Supplementary material**

**Appendix A: search strategy on 21-01-2021**

**21-1-2021:**

| Databases: |  |  |
| --- | --- | --- |
| PubMed, Embase (Ovid), Cochrane CENTRAL | With duplicates | Without duplicates |
| Totaal | 2183 | 1836 |

**PubMed**

570 hits:

("Arthritis, Infectious"[Mesh] OR septic arthritis[tiab] OR sepsis arthritis[tiab] OR bacterial arthritis[tiab] OR septic artritis[tiab] OR sepsis artritis[tiab] OR bacterial artritis[tiab] OR infectious arthritis[tiab] OR infectious artritis[tiab])

AND  ("Treatment Failure"[Mesh] OR "Reoperation"[Mesh] OR "Recurrence"[Mesh] OR "Debridement"[Mesh] OR failure*[tiab] OR reoperat*[tiab] OR recurren*[tiab] OR repeat surger*[tiab] OR revision surger*[tiab] OR surgical revision*[tiab] OR joint revision*[tiab] OR postoperative complication*[tiab] OR orthopedic complication*[tiab] OR reinfection*[tiab]) AND

("Joints"[MeSH Terms] OR joint*[tiab]) and ("Risk Factors"[Mesh] OR "Prognosis"[Mesh:NoExp] OR "Predictive Value of Tests"[Mesh] OR "Probability"[Mesh] OR "Controlled Clinical Trial" [Publication Type] OR "Cohort Studies"[Mesh] OR predict*[tiab] OR risk*[tiab] OR probability[tiab] OR prognos*[tiab] OR score[tiab] OR scores[tiab] OR scoring system*[tiab]) NOT ("Letter"[Publication Type] OR "Editorial"[Publication Type] OR "Comment"[Publication Type] OR "Case Reports" [Publication Type] OR letter[ti] OR editorial[ti] OR comment[ti] OR case report*[ti]) NOT (("Animals"[Mesh] OR "Animal Experimentation"[Mesh] OR "Models, Animal"[Mesh] OR rat[tiab] OR rats[tiab] OR mice[tiab] OR mouse[tiab] OR dog[tiab] OR dogs[tiab] OR pig[tiab] OR pigs[tiab] OR cow[tiab] OR cows[tiab] OR monkey[tiab] OR monkeys[tiab] OR horse[tiab] OR horses[tiab]) NOT ("Humans"[Mesh] OR human*[tiab]))

**Database(s): Embase Classic+Embase 1947 to 2021 January**
Search Strategy:

| **#** | **Searches** | **Results** |
| --- | --- | --- |
| 1 | exp infectious arthritis/ | 23571 |
| 2 | (septic arthritis or sepsis arthritis or bacterial arthritis or septic artritis or sepsis artritis or bacterial artritis or infectious arthritis or infectious artritis).ti,ab,kw. | 8752 |
| 3 | 1 or 2 | 25316 |
| 4 | exp treatment failure/ or reoperation/ or recurrent infection/ or reinfection/ or surgical debridement/ or debridement/ or postoperative complication/ or peroperative complication/ | 656890 |
| 5 | (failure* or reoperat* or recurren* or postoperative complication* or orthopedic complication*).ti,ab,kw. | 2090363 |
| 6 | (revision* adj3 joint*).ti,ab,kw. | 813 |
| 7 | ((repeat* or revision*) adj3 (surger* or surgical*)).ti,ab,kw. | 35940 |
| 8 | 4 or 5 or 6 or 7 | 2500168 |
| 9 | exp joint/ | 524731 |
| 10 | joint*.ti,ab,kw. | 469915 |
| 11 | 9 or 10 | 820249 |
| 12 | risk factor/ or prognosis/ or prediction/ or predictive value/ or predictive validity/ or exp controlled clinical trial/ or cohort analysis/ or retrospective study/ or prospective study/ or follow up/ | 5404173 |
| 13 | (predict* or risk* or probability or score or scores or scoring system*).ti,ab,kw. | 6209160 |
| 14 | 12 or 13 | 8943582 |
| 15 | 3 and 8 and 11 and 14 | 2198 |
| 16 | limit 15 to conference abstract status | 334 |
| 17 | 15 not 16 | 1864 |
| 18 | letter/ or editorial/ or note/ or case report/ or conference paper/ or (letter or comment or editorial or case report).ti. | 5757694 |
| 19 | 17 not 18 | 1562 |
| 20 | (exp animal/ or exp animal experiment/ or exp animal model/ or (rat or rats or mice or mouse or dog or dogs or pig or pigs or cow or cows or monkey or monkeys or goat or goats or horse or horses).ti,ab,kw.) not (human/ or human*.ti,ab,kw.) | 5688081 |
| 21 | 19 not 20 | 1532 |

[Cochrane Central Register of Controlled Trials](https://www.cochranelibrary.com/)

Issue 1 of 12, January 2021

ID Search Hits

#1 (septic arthritis or sepsis arthritis or bacterial arthritis or septic artritis or sepsis artritis or bacterial artritis or infectious arthritis or infectious artritis):ti,ab,kw 701

#2 (joint*):ti,ab,kw 36402

#3 (treatment failure OR debridement OR failure* OR reoperat* OR recurren* OR repeat surger* OR revision surger* OR surgical revision* OR joint revision* OR postoperative complication* OR orthopedic complication* OR reinfection*):ti,ab,kw 219505

#4 #1 and #2 and #3 in Trials 79

**Appendix B: additional details on quality assessment**

The QUIPS tool guides quality assessment in 6 domains: study participation, study attrition, prognostic factor measurement, outcome measurement, study confounding, and statistical analysis and reporting. Risk of bias is reported as low, moderate, or high for each domain and then an overall risk of bias is assigned based on the ratings in each domain. When we judged four or more of the six QUIPS domains to be at low risk of bias, we classified the overall risk of bias as low; when we judged one high risk of bias, with at least one moderate risk of bias, we classified the overall risk of bias as moderate, and when we judged at least 2 high risk of bias or four or more moderate risk of bias, we classified the overall risk of bias as high. See table B2 below.

Table B1: Quality assessment of the individual studies using QUIPS-tool

| **Study ID** | **Study participation** | **Study Attrition** | **Prognostic factor measurement** | **Outcome measurement** | **Study Confounding** | **Statistical analysis and reporting** | **Overall rating** |
| --- | --- | --- | --- | --- | --- | --- | --- |
| Abdou 2019 | Low | Low | Low | Low | High | Low | Low |
| Aim 2015 | Moderate | High | Low | Low | High | Low | High |
| Al-Nammari 2007 | Low | Low | Low | Low | High | Low | Low |
| Assencao 2018 | Low | Unknown | Low | Low | Moderate | Low | Low |
| Besnard 2018 | Low | Low | Low | Low | Moderate | Low | Low |
| Bohler 2016 | Low | Unknown | Low | Low | Moderate | Moderate | Moderate |
| Bohler 2017 | Low | Unknown | Moderate | Low | High | Low | Moderate |
| Bovonratwet 2019 | Low | Low | Low | Low | Moderate | Moderate | Low |
| Bovonratwet 2017 | Low | Low | High | Low | Moderate | Moderate | Low |
| Cho 2016 | Moderate | Unknown | Moderate | High | High | Moderate | Moderate |
| Faour 2019 | High | Unknown | High | Low | Moderate | Low | High |
| Hunter 2015 | Low | Low | Low | Low | Moderate | Moderate | Low |
| Jaffe 2017 | Moderate | Unknown | Low | Low | Low | Moderate | Moderate |
| Jeon 2006 | Moderate | Unknown | Low | High | High | High | High |
| Jiang 2017 | Low | Unknown | High | Low | Moderate | Low | Moderate |
| Johns 2017 | Low | Low | Low | Low | Low | Low | Low |
| Joo 2019 | Low | Low | Low | Low | Moderate | Moderate | Low |
| Jung 2017 | Low | Low | Low | Low | Low | Moderate | Low |
| Kang 2018 | Moderate | Low | High | Low | Moderate | Moderate | Moderate |
| Kao 2019 | Low | Low | Low | Low | High | Moderate | Moderate |
| Khazi 2019 | Moderate | Low | High | Low | Moderate | Moderate | Moderate |
| Khazi 2020 | Low | Low | Moderate | Low | Moderate | Moderate | Moderate |
| Kuo 2019 | Low | Low | Low | High | High | Moderate | High |
| Lee 2019 | Moderate | Low | Low | Low | Moderate | Moderate | Moderate |
| Mabille 2020 | Low | Low | Low | Low | Moderate | High | Moderate |
| Matshuhashi 2011 | Moderate | Low | Moderate | Moderate | Moderate | Moderate | High |
| Rhee 2020 | Low | Low | Low | Low | High | Moderate | Moderate |
| Sammer 2009 | Low | Low | Low | Low | Low | Moderate | Low |
| Stake 2020 | Low | Low | Low | Low | Moderate | Low | Low |
| Stutz 2000 | Low | Low | Low | Low | High | Moderate | Moderate |

| **Table B2: Number of domains out of the total 6 domains in each category** | | | **OVERALL RISK OF BIAS** |
| --- | --- | --- | --- |
| **Low** | **Moderate/Unknown** | **High** |  |
| **6** | **0** | **0** | **LOW RISK** |
|  |  |  |  |
| **4 or 5** | **1 or 2** | **0** |  |
|  |  |  |  |
| **3** | **3** | **0** | **MODERATE RISK** |
|  |  |  |  |
|  | **1** | **1** |  |
|  |  |  |  |
|  |  | **2 or more** | **HIGH RISK** |
|  |  |  |  |
|  | **4 or more** |  |  |
|  |  |  |  |

**B3** **guidelines by Furlan et al.^1^**

- Strong evidence (i.e. consistent (≥75%) findings amongst multiple (≥2) high-quality studies
- Moderate evidence (i.e. findings in one high-quality study and consistent (>75%) findings in ≥2 low-quality studies)
- Limited evidence (i.e. findings in one high-quality study or consistent findings in ≥3 low-quality studies)
- Inconclusive evidence (i.e. findings found in <3 low-quality studies)
- Conflicting evidence (i.e. <75% of the studies reported consistent findings)

^1^Furlan AD, Pennick V, Bombardier C, van Tulder M, Editorial Board CBRG. 2009 updated method guidelines for systematic reviews in the Cochrane Back Review Group. *Spine (Phila Pa 1976)*. *2009*;34(18):1929-41.

**Appendix C**

**Table C1:** Number per joint

| **Number per joint** | |
| --- | --- |
| **Location** | **Number** |
| Shoulder | 6013 |
| Knee | 1814 |
| Hip | 496 |
| Wrist | 42 |
| Elbow | 20 |
| Ankle | 16 |
| Not reported joints | 185 |

**Table C2:** Factors not associated with failure of a single surgical debridement in bivariate analysis

| **Prognostic Factors** | **Number of patients** | **Number significance/total number** |
| --- | --- | --- |
| **Patient characteristics** | | |
| Age | 463 | 0/6 |
| Body Mass Index (BMI) | 371 | 0/5 |
| Smoking | 294 | 0/4 |
| Weight | 59 | 0/1 |
| Length | 59 | 0/1 |
| Alcohol | 51 | 0/1 |
| **Clinical symptoms** | | |
| Duration of prodromal symptoms | 257 | 0/3 |
| Fever | 191 | 0/2 |
| Lateralisation | 97 | 0/1 |
| Large joint | 128 | 0/1 |
| **Serum parameters** | | |
| C-Reactive Protein (CRP) | 417 | 0/5 |
| Red blood cell (RBC) | 63 | 0/1 |
| Mean Corpuscular Volume (MCV) | 63 | 0/1 |
| Haematocrit (Hc) | 63 | 0/1 |
| Haemoglobin (Hb) | 63 | 0/1 |
| Prothrombin time (PT) | 63 | 0/1 |
| International Normalized Ratio (INR) | 63 | 0/1 |
| Partial Thromboplastin Time (PTT) | 63 | 0/1 |
| Glucose | 63 | 0/1 |
| Erythrocyte Sedimentation Rate | 160 | 0/2 |
| Creatinine | 63 | 0/1 |
| Albumin | 63 | 0/1 |
| Blood culture | 191 | 0/2 |
| **Synovial fluid parameters** | | |
| Presence of crystals in synovial fluid | 191 | 0/2 |
| Synovectomy | 52 | 0/1 |
| **Comorbidities** | | |
| History of insulin use | 191 | 0/2 |
| Human Immunodeficiency Virus (HIV) positive | 191 | 0/2 |
| History of coronary artery disease | 191 | 0/2 |
| History of cancer | 179 | 0/2 |
| History of Rheumatoid Arthritis | 129 | 0/2 |
| Hypertension | 160 | 0/2 |
| History of Sickle cell disease | 128 | 0/1 |
| Chronic Obstructive Pulmonary Disease (COPD) | 63 | 0/1 |
| Congestive heart failure | 63 | 0/1 |
| History of invasive joint surgery | 52 | 0/1 |
| History of Chronic Kidney Disease | 51 | 0/1 |
| **Hospital course** | | |
| Recent arthroscopy | 191 | 0/2 |
| Gachter stages | 135 | 0/2 |
| Gachter stadium 1 | 46 | 0/1 |
| Gachter stadium 2 | 46 | 0/1 |
| Kellgren and Lawrence | 59 | 0/1 |
| Length of surgery | 59 | 0/1 |
| Admitted Intensive Care Unit (ICU) | 63 | 0/1 |
| Antibiotic pre-isolated bacteria | 52 | 0/1 |
| UCLA/ASES/SSV scores | 32 | 0/1 |
| Lateralisation | 97 | 0/1 |
| Rotator tear | 97 | 0/1 |
| Time to surgery | 220 | 0/4 |
| Causative organism | 194 | 0/3 |
| Aetiology of infection | 105 | 0/2 |
| Orthopaedic Complications | 27 | 0/1 |
| Without erosions in X-ray and MRI | 57 | 0/1 |
| UCLA = University of California at Los Angeles Shoulder Score, ASES = American | | |
| Shoulder and Elbow Surgerons Shoulder Score , SSV = Subjective Shoulder Score | | |

**Appendix D**


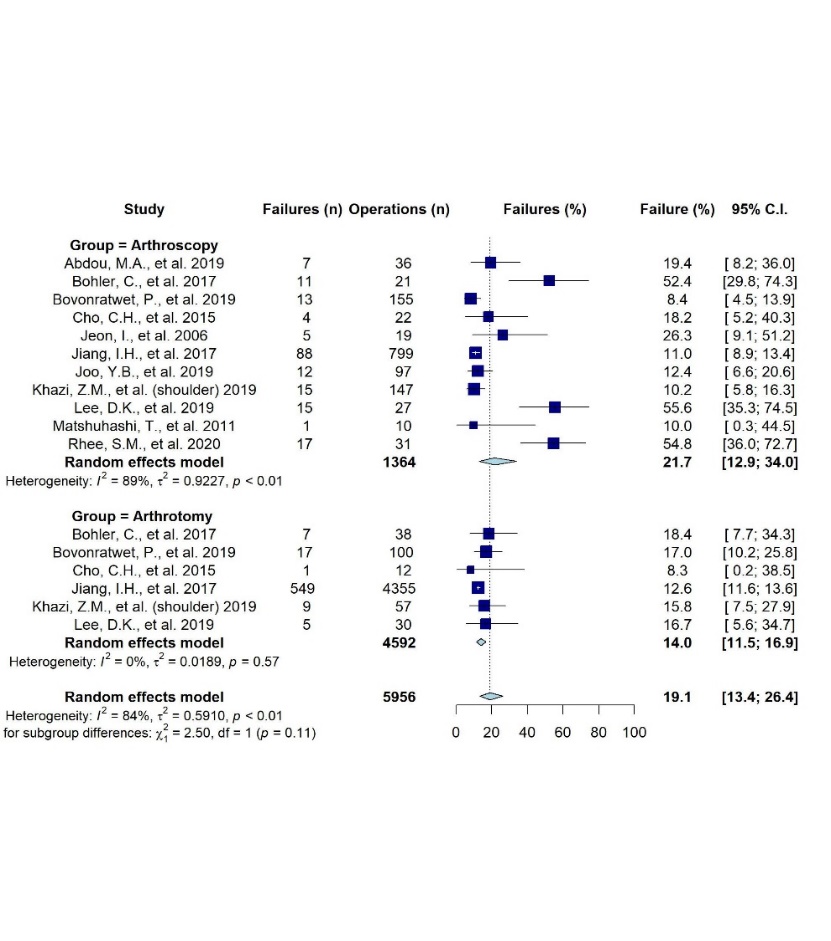
**Figure D1:** Forest plot of the failure rate of the shoulder


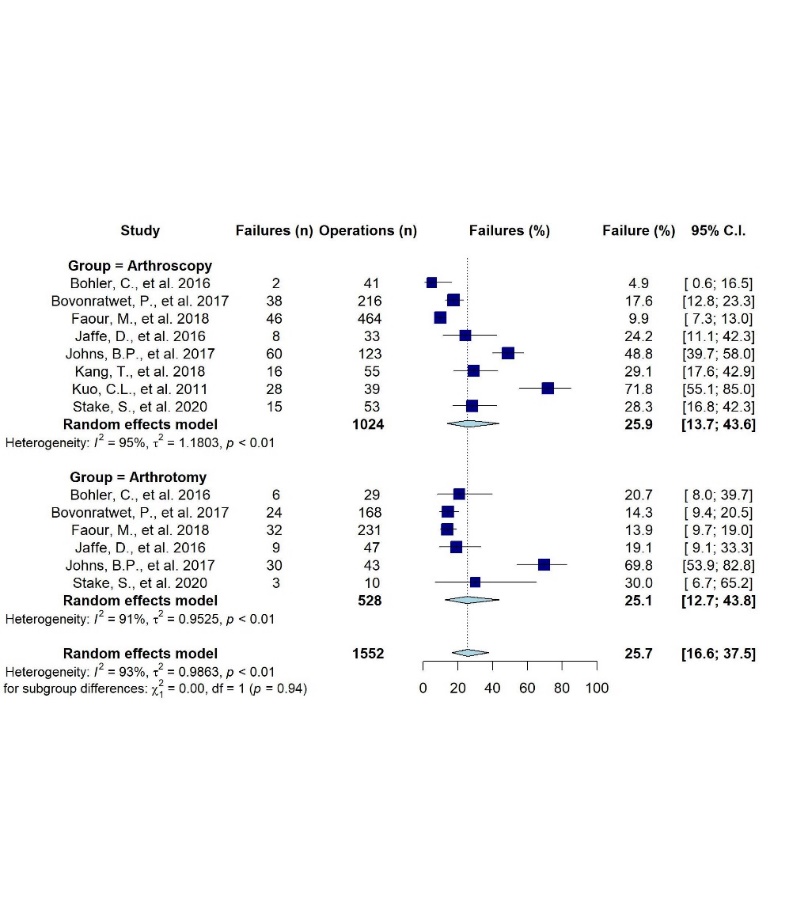
**Figure D2:** Forest plot of the failure rate of the knee
